# Tetanus Severity Classification in Low-Middle Income Countries through ECG Wearable Sensors and a 1D-Vision Transformer

**DOI:** 10.3390/biomedinformatics4010016

**Published:** 2024-01-19

**Authors:** Ping Lu, Zihao Wang, Hai Duong Ha Thi, Ho Bich Hai, VITAL Consortium, Louise Thwaites, David A. Clifton

**Affiliations:** 1 Department of Engineering Science, University of Oxford, Oxford OX1 3PJ, UK; 2 Oxford University Clinical Research Unit, Ho Chi Minh City 700000, Vietnam; 3 Hospital for Tropical Diseases, Ho Chi Minh City 700000, Vietnam; 4 Centre for Tropical Medicine and Global Health, University of Oxford, Oxford OX3 7LG, UK; 5 Oxford Suzhou Centre for Advanced Research, Suzhou 215123, China

**Keywords:** tetanus, electrocardiogram, classification, Transformer, Vision Transformer

## Abstract

Tetanus, a life-threatening bacterial infection prevalent in low- and middle-income countries like Vietnam, impacts the nervous system, causing muscle stiffness and spasms. Severe tetanus often involves dysfunction of the autonomic nervous system (ANS). Timely detection and effective ANS dysfunction management require continuous vital sign monitoring, traditionally performed using bedside monitors. However, wearable electrocardiogram (ECG) sensors offer a more cost-effective and user-friendly alternative. While machine learning-based ECG analysis can aid in tetanus severity classification, existing methods are excessively time-consuming. Our previous studies have investigated the improvement of tetanus severity classification using ECG time series imaging. In this study, our aim is to explore an alternative method using ECG data without relying on time series imaging as an input, with the aim of achieving comparable or improved performance. To address this, we propose a novel approach using a 1D-Vision Transformer, a pioneering method for classifying tetanus severity by extracting crucial global information from 1D ECG signals. Compared to 1D-CNN, 2D-CNN, and 2D-CNN + Dual Attention, our model achieves better results, boasting an F1 score of 0.77 ± 0.06, precision of 0.70 ± 0. 09, recall of 0.89 ± 0.13, specificity of 0.78 ± 0.12, accuracy of 0.82 ± 0.06 and AUC of 0.84 ± 0.05.

## 1 Introduction

Tetanus, a life-threatening infectious disease caused by the bacterium Clostridium tetani, is most prevalent in low- and middle-income countries (LMICs). Although the disease occurs in high-income countries, it is most prevalent in low- and middle-income countries. The disease is common in settings characterised by poor hygiene, limited access to health care and inadequate immunisation programmes [1–3]. The lack of advanced medical equipment and health workers poses challenges in the management of complications of tetanus, such as autonomic nervous system dysfunction (ANSD) and laryngeal spasms, resulting in increased mortality rates, as discussed in [4].

The tetanus toxin disrupts signalling at synapses in the central nervous system, causing agonising muscle spasms and rigidity. In severe cases, its effects on the autonomic nervous system (ANS) can cause cardiovascular instability. Approximately 50% of patients progress to severe disease within 2 to 5 days, and if left untreated, these muscle spasms can affect breathing, requiring the use of powerful muscle relaxants and mechanical ventilation. In mechanically ventilated facilities, this ANS dysfunction is the leading cause of mortality in tetanus patients. However, effective management of this condition remains a major challenge. Early detection of severe tetanus is of paramount importance, as it allows timely intervention and optimises resource allocation [5].

In clinical settings with high patient volumes or limited staff experience, achieving accurate classification can be a daunting task. Advanced continuous monitoring systems and the presence of sufficient health workers in high-income countries have been associated with improved outcomes for patients with tetanus [6,7].

In many resource-limited settings, the availability of close monitoring and timely emergency intervention is typically limited to high-acuity wards or intensive care units, as these facilities have the staff and equipment to provide such services. This increased demand for intensive care in LMICs places an additional burden on already limited resources and may ultimately lead to poorer outcomes for individuals requiring such specialised care [6,8,9]. In addition, a significant number of patients in LMICs, such as Vietnam, are burdened with out-of-pocket medical expenses. As a result, the additional costs associated with ICU care are significantly higher than those associated with standard ward care. Existing research has provided insights into the direct medical expenditure for ICU patients with tetanus, dengue and sepsis in Vietnam [6,8,9].

In resource-limited settings, the use of low-cost wearable sensors is emerging as a promising alternative for tetanus case management. These wearable sensors are wireless, compact and lightweight. Their primary function is to provide real-time, continuous monitoring of vital signs, with the overall goal of enabling early detection of patient deterioration [6,10]. Our previous research has highlighted that the use of ECG monitoring alone may be sufficient to classify the severity of tetanus [11,12]. It is worth noting, however, that the practical implementation of affordable wearable sensors still faces challenges, mainly due to inherent inaccuracies in the continuous physiological data they collect. These inaccuracies arise mainly from data gaps and the significant noise introduced by various factors, thereby undermining their reliability [6].

Our previous studies have investigated the improvement of tetanus severity classification using ECG time series imaging. Our aim in this study is to investigate an alternative method using ECG data without relying on time series imaging as an input, with the aim of achieving comparable or improved performance. This study employs ECG data obtained from wearable sensors utilised in an ICU in Vietnam and suggests a rapid triage tool, developed through deep learning techniques, to categorise tetanus severity based on the Ablett score. We choose a 1D-Vision Transformer to extract the global features of the ECG. The proposed 1D-Vision Transformer outperforms the previous 1D and 2D Convolution Neural Network (CNN) and 2D CNN with Dual Attention mechanisms.

This study provides the following contributions:

- We present a 1D-Vision Transformer model equipped with a self-attention mechanism that enables it to evaluate and assign importance to elements within the input ECG time series data while processing each specific element.
- This is the first time that a 1D Transformer-based method has been investigated to classify the severity of tetanus in LMICs. The proposed 1D-Vision Transformer outperforms the performance of the state-of-the-art 1D and 2D CNN methods in tetanus classification. It promises to improve clinical decision making in resource-constrained settings.
- We illustrate the relationship between the ECG signal and the proposed AI model’s decision using attention scores, showing how the signal exerts varying degrees of influence through different weights.

## 2 Related Work

The healthcare landscape is being transformed by artificial intelligence [13–19]. In traditional machine learning (ML) methodologies, manual feature extraction is often required. For instance, datasets may necessitate the manual extraction of RR intervals, as exemplified in [20]. Support Vector Machines (SVMs) have been employed to automatically gauge the degree of autonomic nervous system (ANS) dysfunction in tetanus patients, as detailed in [21]. However, it is worth noting that deep learning (DL) methods have exhibited superior performance compared to conventional ML techniques like SVMs, as highlighted in [22].

Transformers represent a remarkable advance in the field of computer vision and image analysis [12,23–27]. The field of 2D deep learning with time series imaging has been actively explored for tetanus severity classification, as evidenced by our previous works [11,12,28]. Our previous studies have investigated the improvement of tetanus severity classification using ECG time series imaging. In our previous study [11], we introduced a two-dimensional (2D) convolutional neural network (CNN) augmented with a channel-wise attention mechanism for binary ECG signal classification. Lu et al. [12] proposed a groundbreaking hybrid CNN-Transformer model for tetanus severity level classification using a wearable ECG. This innovative model combines the ability to capture local features via CNN and global features via the Transformer architecture. The input to this model consists of time series images specifically derived from one-dimensional ECG signals using a spectrogram representation. They designed a square time series image format that serves as a bridge between biomedical signals and advanced computer vision algorithms. In addition, Lu et al. [28] introduced a 2D-WinSpatt-Net, a novel Vision Transformer that incorporates both local spatial window self-attention and global spatial self-attention mechanisms. This is the first time that continuous wavelet transform (CWT) has been used for the representation of tetanus ECG information in the form of time series images. This innovative approach resulted in improved tetanus severity classification accuracy, even with shorter tetanus ECG signals of only 20 s. This is a remarkable achievement, especially when compared to the 60 s and 5 min ECG recordings commonly used for heart rate variability.

## 3 Method

**Figure 1:**
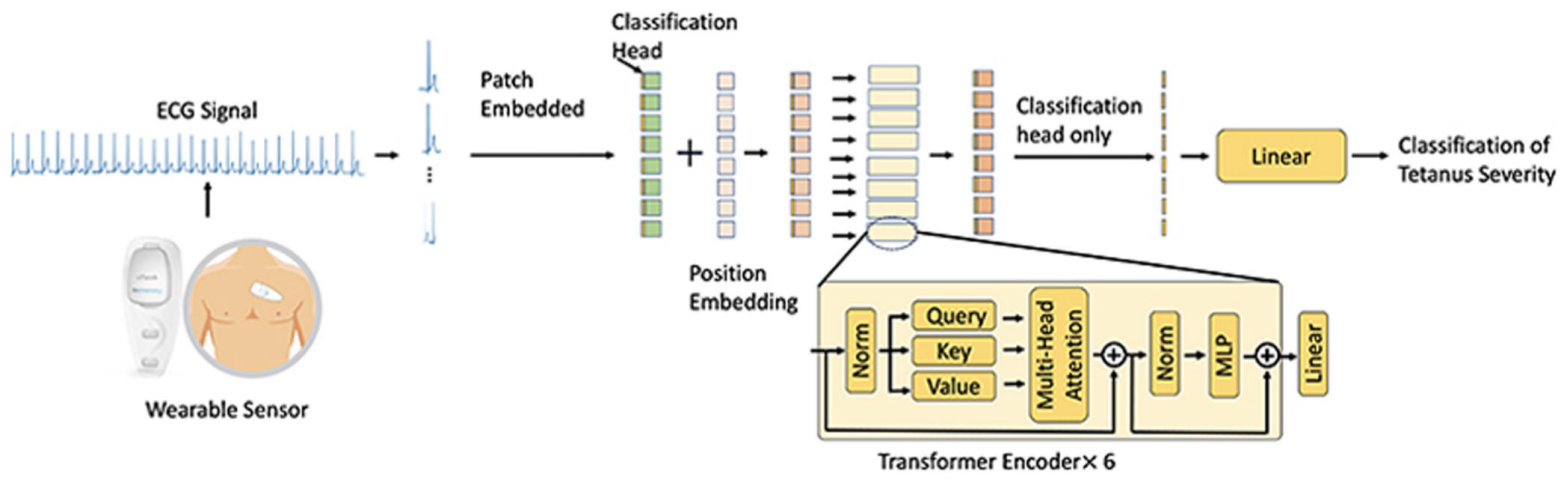
Overview of the tetanus severity classification framework using the proposed method.

The proposed framework includes both data pre-processing and feature extraction using a 1D-Vision Transformer. Figure 1 provides an overview of the framework and illustrates its role in tetanus severity classification using the 1D-Vision Transformer method.

### 3.1 Data Pre-Processing

During the initial data processing phase, our primary goal was to remove noise from the ECG signal. There are two main types of noise that can interfere with ECG signal analysis, as described in [29], low-frequency and high-frequency noise. We acquired single-lead ECG signals using an low-cost, portable monitoring device. To improve data quality, we used a Butterworth filter to remove unwanted noise. The high-pass filter was set to a cut-off frequency of 0.05 Hz, and the low-pass filter was set to a cut-off frequency of 100 Hz. We implemented this data pre-processing step using the SciPy package, as described in [30].

### 3.2 1D-Vision Transformer

We segmented the ECG signal data, denoted as **a**, into flattened non-overlapping patches, represented as ${\tilde{a}}_{p}\in R^{N\times \left(P^{2}\times C\right)}$. Here, *C* represents the number of channels, *N* corresponds to the total number of patches $\left(N=\frac{H}{P}\times \frac{W}{P}\right)$, and *P* indicates the patch size. Subsequently, we transformed these patches into a D-dimensional embedding space through a trainable linear projection. To preserve the spatial information of these extracted patches, we combined the position embeddings with patched embeddings, as outlined below.

(1) $$c_{0}=\left[{\tilde{a}}_{p}^{1}E;{\tilde{a}}_{p}^{2}E;\ldots ;{\tilde{a}}_{p}^{N}E\right]+E_{pos},$$

where $\text{E}\in {\text{R}}^{\left(P^{2}\times C\right)\times D}$ represents the projected patch embedding, and **E**_*pos*_ ∈ R^*N*×*D*^ stands for the learnable position embedding.

After creating the embeddings, we proceeded to apply *L* Transformer layers. Within each Transformer layer, as described in [31,32], three principal components were noted: Multi-Head Self-Attention (MSA), Multi-Layer Perceptron (MLP), and Layer Normalisation (LNorm). The resulting output for the *l*-th layer can be expressed as follows:

(2) $$c_{l}^{\prime }=MSA\left(LNorm\left(c_{l-1}\right)\right)+c_{l-1},\;l=1,\ldots ,L,$$

(3) $$c_{l}=MLP\left(LNorm\left(c_{l}^{\prime }\right)\right)+c_{l}^{\prime },\;l=1,\ldots ,L.$$

Multi-Head Self-Attention

The input matrix **m** ∈ R^*n*×*d*^ was subjected to a transformation, resulting in the generation of three distinct vectors: queries $Que\;\in {\text{R}}^{n\times d_{k}}$, keys $Key\;\in {\text{R}}^{n\times d_{k}}$, and values $\;Val\;\in {\text{R}}^{n\times d_{v}}$. Here, *d*_*k*_ denotes the dimensions of the queries and keys, while *d*_*v*_ represents the dimensions of the values. The mechanism of the scaled dot-product attention, as elucidated in [31], can be expressed through the following equation:

(4) $$Att(\;Que,Key,Val\;)=softmax\left(\frac{\;QueKeyi^{T}}{\sqrt{d_{k}}}\right)\;Val,\;$$

Here, the term $\frac{1}{\sqrt{d_{k}}}$ acts as a scale factor, serving to maintain stable gradients by preventing the softmax function from venturing into regions where gradients become excessively small.

The Multi-Head Self-Attention (MSA) constitutes a fundamental component within the Transformer architecture. It comprises *n* parallel self-attention (SA) heads, each of which dissects the **Que, Key**, and **Val** matrices into distinct subspaces, concurrently executing the scaled dot-product attention operation. Subsequently, the outputs from each head are concatenated and transformed into the final MSA output through a linear projection. The corresponding formula is presented as follows:

(5) $$MSA\;(\;Que,Key,Val\;)=\;Concatenate\;\left(\;Headi_{1},\ldots ,\;Headi_{h}\right)W^{0},$$

(6) $$Head_{i}=Att\left(QueW_{i}i^{Q},Key_{i}i^{K},Val_{i}i^{V}\right),$$

where *W*^*o*^ denotes the multi-headed trainable parameter weights.

## 4 Experiments

### 4.1 Recording ECG Data in Tetanus Patients

The dataset was collected from patients with tetanus admitted to the Hospital for Tropical Diseases, situated in Ho Chi Minh City, Vietnam [6]. In our research, we used ECG data collected from people who had been diagnosed with tetanus. We used the ePatch V.1.0, a low-cost portable monitor manufactured by BioTelemetry, Malvern, PA, USA, as our monitoring device (see Figure 1). The ePatch, which weighs 7 g (ePatch information is available at https://www.philips.co.uk/healthcare/resources/landing/epatch, accessed on 8 January 2024) was securely attached to the patient’s chest skin to ensure reliable adhesion. The device records two channels of ECG data, and the sampling rate is 256 Hz.

The two channels of the ePatch device (referred to as channel 1 and 2) do not directly correlate with the ECG leads 1 and 2 of the conventional bedside monitor, as mentioned in [12]. Clinical staff used the Ablett scoring system to grade severity as follows: grades 1 and 2 (no or mild spasms) define “mild” disease, and grades 3 and 4 (spasms interfering with respiration with/without autonomic nervous system dysfunction) define “severe” disease. The details of the tetanus data information can be found in [11,12].

### 4.2 Implementation Details

Pre-processing. We extracted 30 ECG time series, each with a duration of 60 s, from every ECG example file. This resulted in a training dataset comprising a total of 4230 ECG time series, with 2370 samples indicative of mild tetanus and 1860 samples indicative of severe tetanus. Our validation dataset consisted of 540 ECG time series (270 mild cases and 270 severe cases), while the test dataset comprised 570 ECG time series (360 mild cases and 210 severe cases). The categorization of mild and severe tetanus cases was performed by clinicians.

Experimental Setup. Based on our experiments, the following selected hyperparameters of the proposed 1D-Vision Transformer achieved optimal results (see Table 1). Each 1-min ECG dataset comprises 15,360 data points. We applied a 1D convolution to the input signal (60-s ECG), producing 384 sets, each containing 320 data points. We then rearranged the original tensor according to the desired order, resulting in a new multidimensional rotated tensor with 320 sets, each containing 384 data points.

The model was trained with the following specifications: 100 epochs using the Adam optimiser, a learning rate of 0.001 and a batch size of 32. The torch.nn.CrossEntropyLossis was selected as the loss function. The implementation of the suggested 1D-Vision Transformer was carried out in Python 3.7 using PyTorch. The experiments were carried out on hardware equipped with the NVIDIA RTX A6000 48 GB GPU.

### 4.3 Baselines

In our study, we performed a comparative analysis between the 1D-Vision Transformer, our proposed method, and three different baseline approaches. These baseline methods included two 2D deep learning techniques introduced by Lu et al. [11] and a 1D-CNN method.

### 4.4 Evaluation Metrics

In this study, we utilised multiple performance metrics to assess the effectiveness of the binary classification task. These metrics were the F1-score, precision, recall, specificity, accuracy [22] and the area under the curve (AUC) [33]. To ensure the reliability of our findings, each model was executed five times, with subsequent computation and reporting of the performance metric averages and standard deviations using an independent test dataset. A higher AUC value serves as an indicator of the model’s superior ability to accurately distinguish between severe and mild cases of tetanus.

## 5 Experimental Results

### 5.1 Data Pre-Processing Analysis

We delved into the realm of pre-processing techniques in ECG analysis, recognising that noise removal at this stage can significantly improve classification performance. Our primary objective was to quantify the positive impact of these pre-processing steps. As shown in Table 2, we observed a significant improvement in F1-score (0.03 increase), precision (0.06 increase), specificity (0.05 increase), AUC (0.03 increase) and accuracy (0.04 increase) following the removal of data noise in the input to our proposed 1D-Vision Transformer. This demonstrates that the application of the Butterworth filter in the pre-processing step effectively removes unwanted noise, resulting in higher quality ECG data as the input to our model. This in turn leads to improved classification accuracy for tetanus severity.

### 5.2 Comparisons

We evaluated the proposed 1D-Vision Transformer by comparing it to three different deep learning techniques, including models using 1D (ECG signal) and 2D (time series image) data as input. In light of the experimental outcomes presented in Table 3, the 1D-Vision Transformer method using ECG (non-imaged data representation) as input achieves the best performance in diagnosing tetanus. The 1D-Vision Transformer outperforms the 1D-CNN.

### 5.3 Interpretable ECG

**Figure 2:**
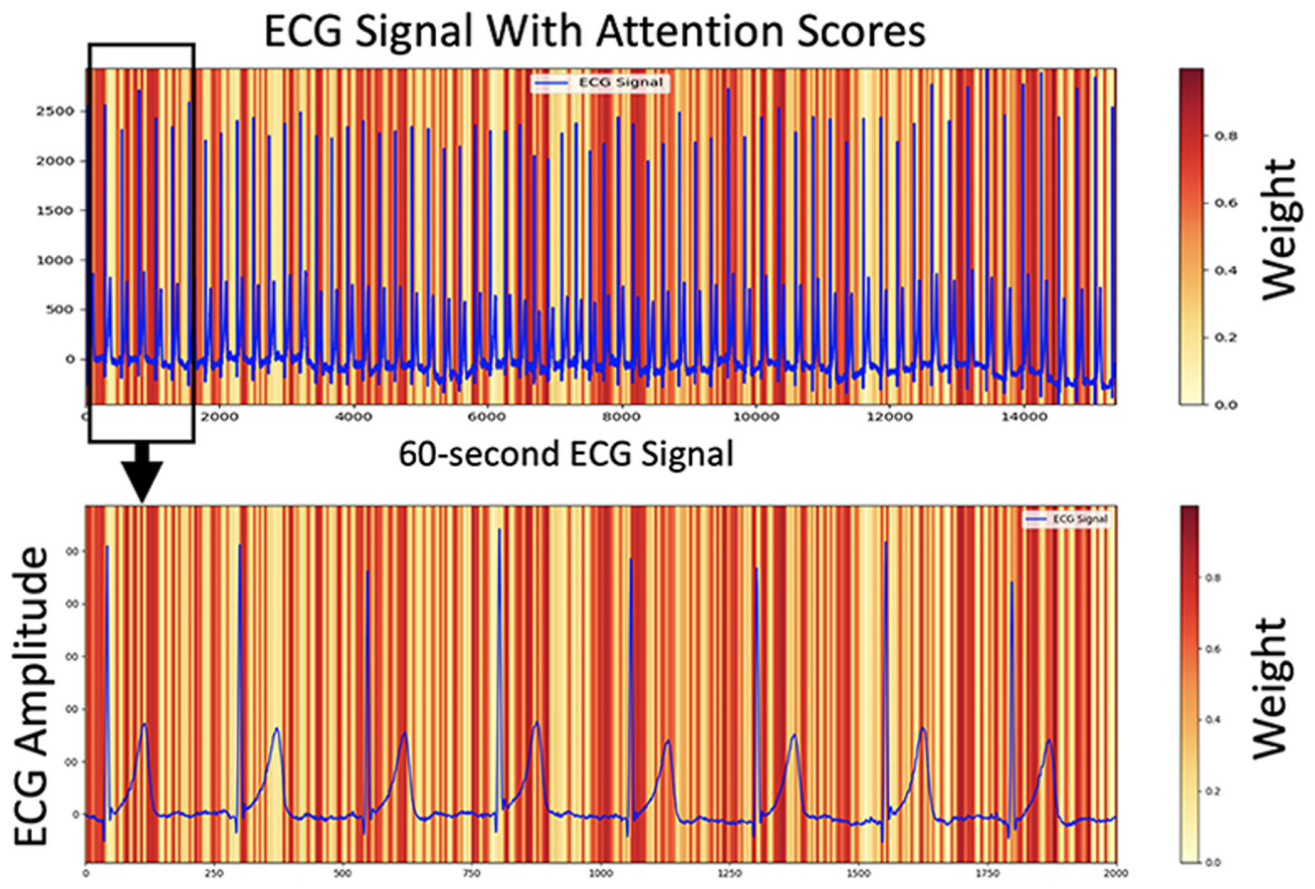
An example ECG waveform with corresponding attention scores: darker red signifies a greater influence on the proposed 1D-Vision Transformer model’s categorisation of mild tetanus.

We used the attention scores to interpret which part of the ECG signal the model is focusing on for the classification of tetanus severity. We represent high scores with a darker shade of red, indicating that the ECG region coloured in darker red has a greater influence on the model’s decision. Figure 2 displays a 60 s ECG example along with the attention scores that the proposed model relies on when categorising mild tetanus.

### 5.4 Misclassification

**Figure 3:**
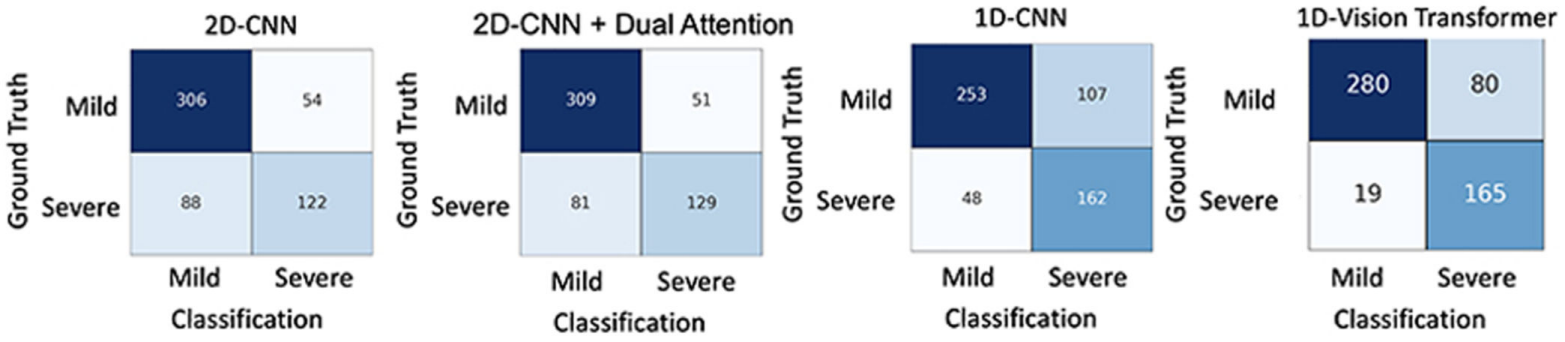
The confusion matrices for tetanus severity classification using different deep learning methods: 2D-CNN and 2D-CNN + Dual Attention with 60 s window log spectrograms as the inputs (without downsampling); and 1D-CNN and 1D-Vision Transformer with 60 s ECG data as the inputs, representing an image-free data representation.

We used the same strategy to generate confusion matrices, as detailed by Lu et al. [11]. The confusion matrices depicted in Figure 3 provide a comprehensive overview of the performance of each method in our experiments as well as the types of misclassifications they exhibit between the mild and severe levels. The successful detection rate for severe tetanus diagnosis reached 165 after employing the 1D-Vision Transformer, which represents the highest accuracy achieved among these deep learning methods. The performance of the 1D-Vision Transformer surpassed that of the 1D-CNN in classifying mild and severe tetanus cases.

## 6 Discussion

The proposed 1D-Vision Transformer is equipped with a self-attention mechanism that enables it to consider the importance of elements in the input ECG time series data when processing a particular element. This allows it to capture global relationships and dependencies within the data. In other words, it can understand how different parts of the input ECG time series data relate to each other, regardless of their position. We enhanced the classification of tetanus severity levels through the utilisation of a 1D deep learning approach, surpassing the performance of 1D-CNN. In our earlier investigations, as detailed in [11,12,28], we discovered that representing 1D ECG as time series images, serving as the input for 2D deep learning methods, yielded superior performance. While the performance of the proposed 1D-Vision Transformer does not surpass that of [11,12,28], it represents a promising first step in exploring the field of 1D deep learning approaches for tetanus severity level diagnosis. Our goal is to improve the 1D-Vision Transformer for classification of mild or severe tetanus in future research efforts. In addition, the 1D-Vision Transformer can serve as a benchmark for our future 1D deep learning approaches. Furthermore, the proposed method can be applied to other biomedical signal analyses, such as sepsis or dengue.

## 7 Conclusions

We have proposed a 1D-Vision Transformer for tetanus severity classification. Our experimental results clearly demonstrate the superiority of our proposed method over other advanced deep learning approaches in the context of tetanus severity classification. This deep learning framework promises to significantly improve clinical decision making and streamline the allocation of limited healthcare resources, particularly in low- and middle-income countries (LMICs). In our future endeavours, we will strive to further enhance the novelty and effectiveness of the 1D-Vision Transformer-based method. Moreover, the versatility of the 1D-Vision Transformer allows its application in various classification tasks, including those involving time series data.

## Figures and Tables

**Table 1:** Employed parameters of the proposed 1D-Vision Transformer.

Parameters		
in_channels	1	the number of channels of the image
patch size	48	the size (resolution) of each patch
num_transformer_layer	6	the number of Transformer blocks
embed_dim	384	the embedding dimension
Mlp_size	1024	the number of neurons in the hidden layer
num_heads	6	the number of heads
mlp_drouppout	0.1	the dropout for the MLP layers
embedding_dropout	0.1	the dropout for the embeddings
num_class	2	the number of classes

**Table 2:** A quantitative analysis of the proposed 1D-Vision Transformer, comparing with and without data pre-processing. The outcomes are presented as the mean ± standard deviation, highlighting the top performance in bold.

1D-Vision Transformer	F1-Score	Precision	Recall	Specificity	Accuracy	AUC
without data pre-processing	0.74 ± 0.04	0.64 ±0.07	**0.89 ± 0.04**	0.73 ± 0.08	0.78 ± 0.05	0.81 ± 0.03
with data pre-processing	**0.77 ± 0.06**	**0.70 ± 0.09**	0.89 ± 0.13	**0.78 ± 0.12**	**0.82 ± 0.06**	**0.84 ± 0.05**

**Table 3:** A quantitative analysis of the proposed 1D-Vision Transformer, compared to baseline methods that employ either 60-s time series image [11] or original 60-s ECG as input. The outcomes are presented as the mean ± standard deviation, highlighting the top performance in bold.

	The Time Series image as the Input					
Method	F1-Score	Precision	Recall	Specificity	Accuracy	AUC
2D-CNN [11]	0.61 ± 0.14	0.68 ± 0.07	0.57 ± 0.19	0.85 ± 0.02	0.75 ± 0.07	0.72 ± 0.09
2D-CNN + Dual Attention [11]	0.65 ± 0.19	**0.71 ± 0.17**	0.61 ± 0.21	**0.86 ± 0.09**	0.76 ± 0.11	0.74 ± 0.13
	**The ECG as the Input**					
**Method**	**F1-Score**	**Precision**	**Recall**	**Specificity**	**Accuracy**	**AUC**
1D-CNN [11]	0.65 ± 0.14	0.61 ± 0.05	0.77 ± 0.25	0.70 ± 0.13	0.73 ± 0.05	0.74 ± 0.08
**Proposed 1D-Vision Transformer**	**0.77 ± 0.06**	0.70 ± 0.09	**0.89 ± 0.13**	0.78 ± 0.12	**0.82 ± 0.06**	**0.84 ± 0.05**

## Data Availability

Data are contained within the article.
